# Supplementary figures and images for: Improved eukaryotic detection compatible with large-scale automated analysis of metagenomes
Source: Microbiome. 2023 Apr 10;11:72. doi: 10.1186/s40168-023-01505-1 (PMC10084625; doi:10.1186/s40168-023-01505-1)

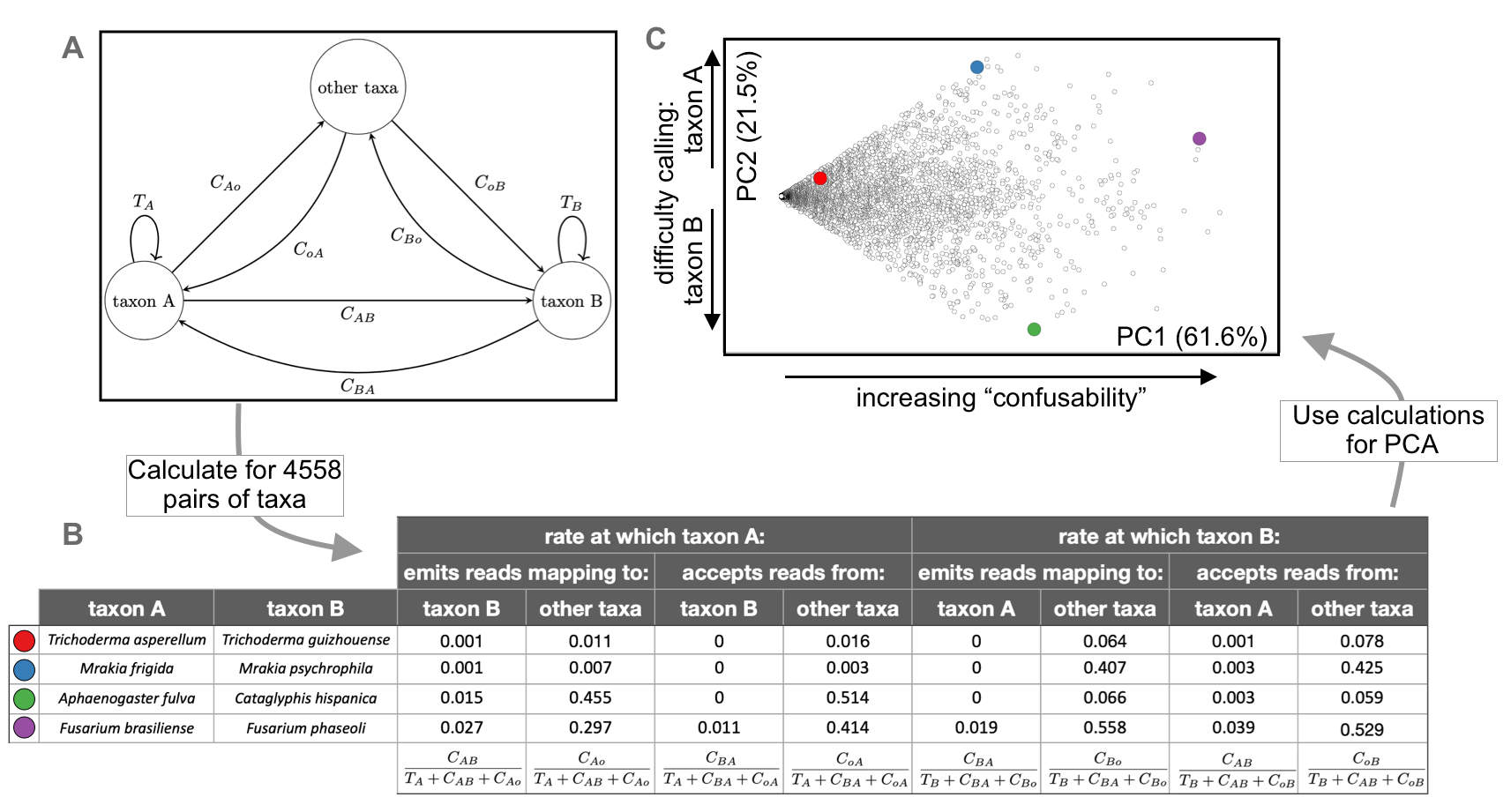

Supplement: Supplementary file 4 — Additional file 3: Supplementary Figure 1. Mathematical framework for evaluating the potential confusability of closely related pairs of eukaryotic species present in the same sample. (A) Schematic showing all eight parameters of the model for relating read mapping within and outside of a given pair of taxa. These eight parameters were calculated for all 4558 pairs of taxa. (B) Table showing four example pairs of taxa, each with different read mapping behavior. (C) Principal component analysis (PCA) plot showing first two principal components from the analysis of the full dataset from panel B. Axes show interpretation of principal components. Color points represent the position of example taxon pairs from panel B. [file 40168_2023_1505_MOESM3_ESM.tiff]

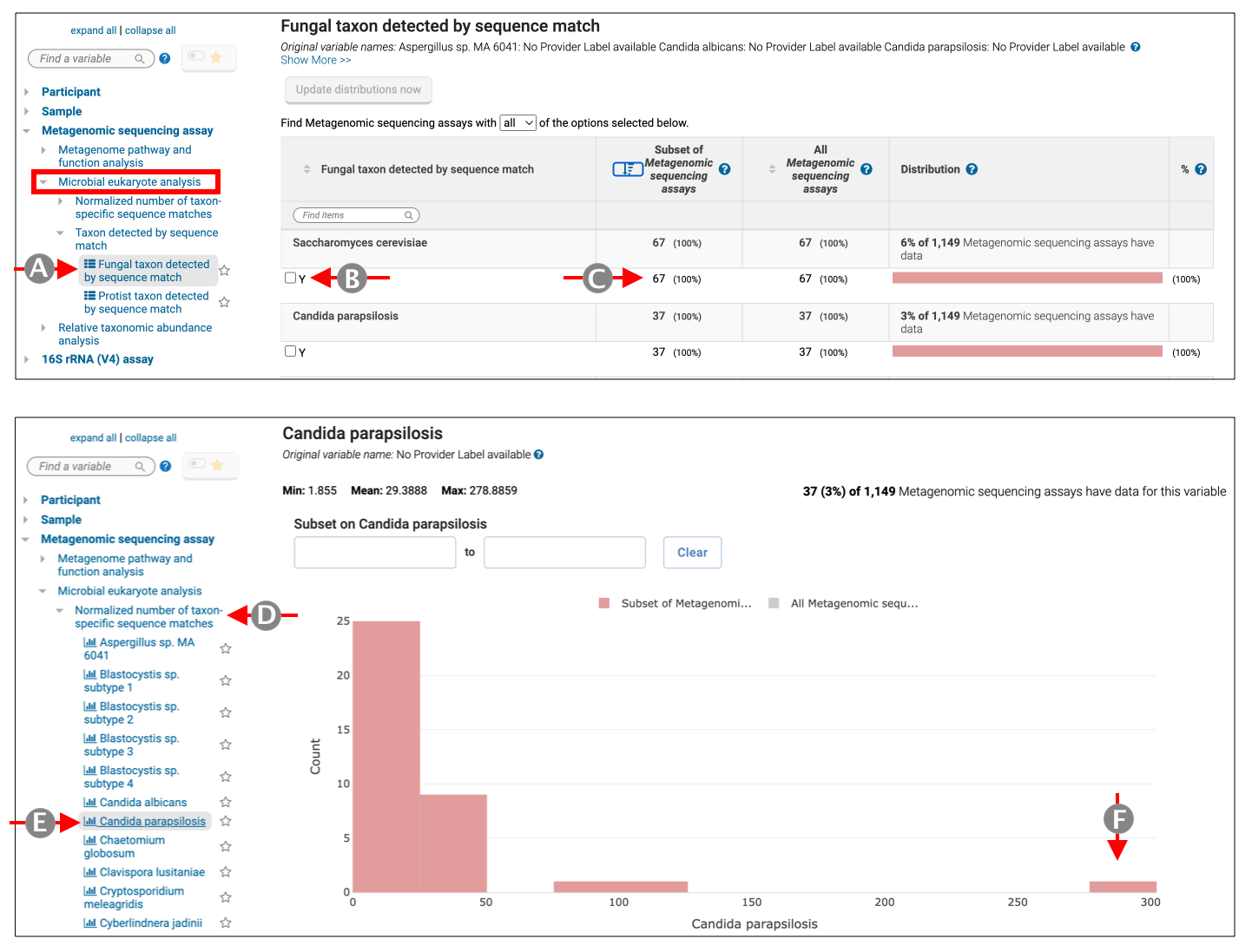

Supplement: Supplementary file 5 — Additional file 4: Supplementary Figure 2. CORRAL results are readily searchable and queryable on MicrobiomeDB.org. Screenshots showing CORRAL results for the DIABIMMUNE study that includes 1149 samples. Results are displayed as detection (top) or quantification (bottom). Users select broad groups of microbial eukaryotes (e.g. (A) fungi) and the select specifc species (B) from a multipick list. Numbers of samples in which the taxon was detected are shown and can be used to filter the dataset (C). For quantification, users select the ‘normalized’ number of sequence matches (D) and then a species of interest (E) to display a histogram of sample count by abundance for the the taxon of interest, making it simple to identify samples with high (F) or low abundance. Users can then select only those samples that contain a specific abundance or fall within an abundance range. [file 40168_2023_1505_MOESM4_ESM.tiff]
